# Promoting a social equity approach to sexual harassment prevention across higher education: the Uni4Equity project

**DOI:** 10.3389/fpubh.2026.1848811

**Published:** 2026-05-29

**Authors:** Carmen Vives-Cases, Marina Berbegal-Bernabeu, Esther Ríos-Albert, Ariadna Cerdán-Torregrosa, Vanesa Pérez-Martínez, Barbara Jankowiak, Sylwia Jaskulska, Sofia Neves, Mafalda Sousa, Joana Topa, Estefânia Silva

**Affiliations:** 1 Department of Community Nursing, Preventive Medicine and Public Health and History of Science, University of Alicante, Alicante, Spain; 2 CIBER of Epidemiology and Public Health (CIBERESP), Instituto de Salud Carlos III, Madrid, Spain; 3 Department of Sociology II, University of Alicante, Alicante, Spain; 4 Faculty of Educational Studies, Adam Mickiewicz University in Poznan, Poznan, Poland; 5 Department of Social and Behavioral Sciences, University of Maia, Maia, Portugal; 6 Interdisciplinary Centre for Gender Studies, University of Lisbon, Lisbon, Portugal

**Keywords:** equity-centered approach, gender-based violence, institutional response, sexual harassment, universities

## Abstract

Sexual harassment (SH) is a pervasive form of gender-based violence that takes place in universities worldwide, undermining individual, community, and institutional integrity. SH occurs in-person and online, and it affects undergraduate, master's and doctoral students, and academic, administrative and service staff. Beyond its documented harm to health and well-being, SH also negatively influences professional and academic trajectories. Although formal institutional resources and mechanisms to prevent and combat SH exist, they remain limited and are not always effectively used. Most victims-survivors manage the situation on their own. Fear of retaliation and power imbalances affect not only victims-survivors, but also bystanders’ actions. Universities must adopt equity-centered strategies that address structural causes, transform institutional cultures, reduce barriers to reporting and access to support, and protect vulnerable groups.

## 1 Introduction

Sexual harassment (SH) is one of the most prevalent forms of gender-based violence in universities (1). It manifests physically, verbally, and digitally (2, 3). Responses from those affected, as well as bystanders, are often shaped by social pressure, sexist norms, and the normalization of these acts, which prevents them from being recognized as violence (3).

Academic culture has been traditionally characterized by relationships that are hierarchical, competitive, and individualized, all of which give rise to abusive situations such as SH (4, 5). In fact, SH is one of the most prevalent forms of gender-based violence in universities (5, 6). Evidence from the UniSAFE project indicates that almost one in three students and staff (31%) report having experienced SH within their institution (6). It is essential to promote approaches that are not limited to sanctioning harassment behaviors, but that fundamentally transform the structures that enable SH. Along these lines, the gender-transformative approach aims to reconfigure gender dynamics, by rethinking existing power relations and redistributing resources, responsibilities and expectations between women and men. This approach seeks to modify the gender norms that perpetuate inequalities, challenging both social imaginaries and the institutional practices that sustain them (7), through the promotion of awareness-raising, collective empowerment and the active participation of the entire university community in building a more egalitarian institution free from violence (8).

Recent European policy developments, such as the Zero-Tolerance Code of Conduct on gender-based violence in the EU research and innovation system (2024) highlight the need for comprehensive and systemic responses to this issue (9). In addition, gender-based violence, including SH, has been explicitly identified as a key area to be addressed within Gender Equality Plans (GEPs), which are a requirement for organizations applying for EU research funding, as outlined by the European Commission Directorate-General for Research and Innovation (2025) (10). In public policy, it is essential to adopt an intersectional perspective in the design of policies and actions to eradicate SH in the university environment. Intersectionality (11) describes how each person’s experience is influenced by the interaction of multiple interconnected systems of privilege and oppression—such as gender, ethnicity, sexual orientation, social class and disability, among others—that generate differing and specific forms of discrimination and vulnerability (3, 12). Incorporating this approach in university strategies would not only contribute to greater inclusion, but it would also allow for institutional responses to be more sensitive, contextualized and effective in addressing the diversity of experiences of people who experience or have experienced SH.

In universities, the risk is shaped by multiple factors that make some groups more vulnerable (13–15). The social determinants of SH illustrate how certain conditions increase the likelihood that SH will occur into three levels: structural, institutional, and individual. The structural level includes systemic determinants present in contemporary societies, such as gender inequalities and other forms of inequality that disproportionately affect women, including the feminization of poverty, wage gaps, underrepresentation in leadership, and coexistence with other forms of violence. At the institutional level, determinants relate to university organization, including the reproduction of inequalities through hierarchical dynamics, persistent invisible barriers such as glass ceilings, and meritocratic logics that overlook unequal starting conditions. The lack of a gender perspective in policies and programs further perpetuates cultures that fail to adequately address SH. At the individual level, behavior is shaped by socially learned attitudes such as sexism, traditional gender roles, harassment myths, impostor syndrome (16), and the Matilda effect (17), influencing both perpetrators’ actions and victims’ ability to identify, report, or resist SH.

Given this context, it is essential to adopt approaches centered on social equity to prevent and address SH in universities. Social equity refers to the absence of social inequalities and their consequences for the well-being and health of the population. The gender-transformative approach and the concept of intersectionality offer solid theoretical frameworks for understanding how power inequalities operate and how different forms of oppression intersect and affect students, faculty and university staff in disparate ways (7, 18, 19). These approaches not only promote gender equality, but also advocate social justice, empowerment, and the valuation of diversity as fundamental pillars of safe and inclusive institutions. Promoting social equity in the university involves designing policies that are sensitive to gender, diversity, and the distribution of power, to build academic spaces that are safe, inclusive, and free from violence.

This brief presents recommendations on how to strengthen universities’ capacity to prevent SH with a social equity approach, based on the findings of Uni4Equity, a project funded by the European Union’s CERV-2022-DAPHNE program (Ref. 101,094,121-Uni4Equity) (20). The project adopted a sequential exploratory design, implemented in three phases: (a) initial diagnosis using surveys, interviews and documentary reviews; (b) the implementation of large-scale interventions at different levels of prevention (primary, secondary, tertiary) and scope of application (interpersonal, institutional, social); and (c) the evaluation of results and overall impact.

## 2 Policy options and implications: challenges to addressing inequalities in SH

### 2.1 Challenge 1. Addressing inequalities in the risk of victimization

The challenges presented are based on quantitative and qualitative data generated within the Uni4Equity project, a multicountry mixed-methods study conducted between 2023 and 2026 across 6 European Universities (University of Alicante, University of Antwerp, University of Maia in Porto, University of Applied Sciences Burgenland in Eisenstadt, Adam Mickiewicz University in Poznań and University of Verona). The project follows an exploratory sequential design combining surveys, semistructured interviews and desk reviews to identify institutional strengths and weaknesses in preventing and responding to online and offline SH at the university. The evidence presented in this policy brief draws from the initial assessment phase of the project, including survey data from more than 7.500 members of the university community and 89 qualitative interviews with key informants. Further methodological details are available in the published study protocol (20).

The results of the Uni4Equity project show that SH affects the entire university community, including undergraduate, master’s and doctoral students, as well as academic, administrative and service staff. However, the distribution of the risk of SH is unequal, and some groups are especially vulnerable because of their social circumstances. Here we list some challenges to consider in the design of prevention strategies related to the different ways in which social inequalities manifest in SH at the university. Challenge 1: addressing inequalities in the risk of victimization

According to data collected from the Uni4Equity survey with a sample of more than 7,500 members of 6 European universities, women had almost twice the probability of experiencing SH compared to men. Just over 30% of women reported having been exposed to some form of SH or inappropriate sexual conduct, compared to 7.9% of men. Similarly, across the 89 interviews conducted by the project with key informants, women are identified as the main victims, especially those in situations of greater social vulnerability marked, for example, by job insecurity, economic precarity, lack or absence of support networks, or having already suffered other forms of violence in the past (such as bullying or intimate partner violence).

“Women… are a part of socially vulnerable groups. For example, there are economic patterns […] or some type of disability […] or they have a history of being bullied.” *- Institutional representative, woman.*

“In general, women are still at great risk, it’s still a central issue.” *- External institutional representative, man.*

Members of the LGBTQIA+ community had almost twice the probability of experiencing SH compared to heterosexual individuals. Among LGBTQIA+ community, bisexual people experienced SH most frequently (50.5%), followed by homosexual people (42%) and asexual people (40%). Cisgender men experienced SH less frequently (29.7%) than trans and intersex people (58.9%). Specifically, LGBTQIA+ phobic beliefs were mentioned in the interviews as an emerging factor that increases the risk of SH against LGBTQIA+ people.

“Students who belong to the LGBT community are already less privileged in one way or another.” *- Student representative, woman.*

“You’re even more vulnerable when you have a sexual orientation that’s different from the norm.” *- External institutional representative, woman.*

Frequently, situations of SH are related to power imbalances. The project interviews reveal the vulnerability of young people, both students and those in lower positions in the academic hierarchy.

“Probably also doctoral students […] I don’t like this hierarchical assessment, but, for example, service workers, who in our structure do not occupy very high positions, might not have the courage to report certain inappropriate behaviors.” *- Institutional representative, woman.*

The lack of social support networks nearly doubles the probability of experiencing SH. Project data indicate that 44% of people lacking social support have suffered SH. For example, interviewees mentioned being an international student and coming from homes with less parental presence as factors contributing to a lack of social support. On the other hand, other situations of vulnerability emerged, such as having a disability or a low socioeconomic status.

“I think of students who come from single-parent families or from families where parents aren’t present much or have a low level of education.” *- Student representative, woman.*

“People with disabilities, of course, any disability, whether physical or psychological. They tend to be vulnerable because harassers perceive them that way.” *- Staff member, woman*.

### 2.2 Challenge 2. Reducing inequalities in access and trust of formal support institutions

Most people who experienced SH said they turned first to informal support networks such as family and friends (6.8%), while a smaller percentage turned to formal supports such as faculty and university resources (1.5%). In line with these data, the interviews show that men tend to trust institutional channels more, while many women turn to informal support networks or self-management strategies (such as blocking or reporting the aggressor on social media or avoiding physical contact), especially when they have had prior negative experiences with official resources.

“The boss simply said: “Look at this,” *opened a drawer and said:* “Here I put all the reports and I don’t even open them.” - *External institutional representative, woman.*

“Online there’s always the option to report it […] or remove the account of the person who posted those things.” *- Student representative, woman.*

Language is among the barriers people face in accessing resources— particularly affecting people from other countries who do not speak the local language fluently. This barrier can manifest in different ways: in-person, due to lack of language skills; or in an online format, when institutional websites provide reporting tools that are not very accessible or visible and are not adapted to different languages, formats or channels. All of this hinders access to support and reporting mechanisms. Furthermore, information is often scattered and not centralized, which can contribute to a lack of knowledge about the process and affects trust and use of resources. For example, most universities do not have a frequently asked questions (FAQ) section that provides clarity about action protocols, which prevents the information and reporting process from being transparent.

There is also a lack of accessible, inclusive and user-friendly language in explanations of how to detect, prevent, respond to and report a case of SH. This is the primary obstacle for people who have sensory disabilities (such as visual or hearing impairments) or developmental disabilities (such as autism). In the case of functional disabilities, architectural barriers often hinder in-person access.

“A person, well, I don’t know, to give you an example, is autistic or has problems communicating. Well, it’s multiple times more difficult. Or if they have any disability, well…” *- Student representative, woman.*

Additional barriers include the lack of flexible office hours (for example, in the case of students who attend classes in the afternoon), access to training and information, and the lack of dissemination of resources in online formats and in-person.

“I often feel that they don’t make it easy for those of us who study in the afternoon.” *- Student representative, woman.*

“The training is great, in the end its super accessible, but it doesn’t reach people (…) to spread the word about all the measures that exist, about everything related to the trainings that are offered.” *- Institutional representative, woman.*

### 2.3 Challenge 3. Improving equity-focused case management of SH in the university

The presence of power imbalances can cause different consequences depending on the profile of the person affected. Managing a case of SH of a doctoral student is not the same as that of a full/associate professor, since the latter may not have as much fear of the consequences; each case is different. This is closely related to the reporting and management of SH cases.

The results of the project (surveys and interviews) suggest that victims of SH face multiple barriers that deter and even prevent formal reporting. In this regard, the survey showed that people feel uncertainty about which behaviors are serious enough to be reported. Indeed, this is the most common barrier among women (54.7%), men (42.7%), students (53.8%) and staff (49.4%). Along the same lines, the interviews suggested that people perceive that only cases considered most visible and serious, such as those involving physical contact, end up being reported.

“I think that maybe only the most serious actions get reported.” *- Staff member, woman.*

Another barrier to reporting mentioned by participants is the fear of revictimization. The survey revealed the university community’s concern about the complexity of the reporting process; there was greater concern among women (9.3%) and staff (8.8%). Interviewees also recognized reporting as a complex and bureaucratic process that can be emotionally draining, which leads victims leaving things as they are and adopting informal alternatives rather than reporting (changing work groups, avoiding the aggressor or turning to informal support networks).

“I wouldn’t be surprised if there are many people—many women—who deal with it, who don’t consider reporting or coming forward. Rather, they look for their ways, informal ways, or… ‘this will pass.” *-Institutional representative, woman.*

There are also conditioning factors, such as economic dependence and fear of professional or academic retaliation. The survey highlights concern about possible retaliation by the aggressor, which was more frequent among women (12.4% compared to 5.9% of men) and staff (13.5% compared to 9.8% of students).

“There are many types of retaliation in terms of professional promotion, academic advancement, etc. And then there is economic dependence; in many cases you can’t take the risk.” *- Staff member, woman.*

However, fear of retaliation and university hierarchical dynamics affect not only victims but also shape the actions of SH bystanders. The interviews revealed that students and staff in lower positions tend not to intervene for fear of negative consequences. Nevertheless, in the survey, women (78.7%) and staff (82%) consistently show slightly higher levels of proactive and preventive behavior than men (66.6%) and students (71.1%), especially in situations that require empathy (asking someone if they need help or staying with someone under the influence of alcohol so they are not left alone) or that involve subtle forms of sexism (sexist comments or jokes).

“There’s also a bit of fear, like, if I say something against this professor now, that could become a problem for my studies.” *- Student representative, man.*

## 3 Actionable recommendations

The actionable recomendations presented in this section are grounded in the findings obtained through the designs, implementation, and evaluation of the Uni4Equity interventions developed across the participating universities. Further details regarding the intervention framework and evaluation strategy are available in the published study protocol (20).

SH at the university requires a comprehensive approach that considers the diversity of the university community, addresses the root causes of SH, and promotes changes at different levels where gender relations occur. Training is an essential tool for promoting these changes, but it must be complemented by strengthening institutional policies, building alliances with key agents, and improving resources and mechanisms for addressing SH and other forms of violence and discrimination. To be effective, actions must take place at three levels of prevention.

### 3.1 Primary prevention: address the social determinants of SH

Primary prevention of SH involves actions directed at the entire university community regarding factors that may facilitate SH. It requires strategies that transform gender relations at the social, organizational and interpersonal levels. Based on the Uni4Equity project, key actions include:

- Increasing awareness campaigns regarding SH and other forms of gender-based violence at the university, using an intersectional approach to define the problem and its causes.
- Addressing the social determinants of SH in training activities for the entire university community, such as workshops aimed at both students and staff.
- Explicitly recognizing power asymmetries and dependency relationships rooted in university hierarchies and academic cultures. These can be addressed through policies, such as equality plans, that tackle gender and social inequalities in academic and professional trajectories, challenge stereotypes, and promote gender-focused and intersectional approaches in teaching and research.

### 3.2 Secondary prevention: prioritize inequalities in access to resources

A key course of action at this level is to reduce inequalities in access to formal support resources and to promote interventions targeted at groups that are more vulnerable to SH and other forms of violence and discrimination. Improving accessibility to formal support resources requires:

- Ensuring that information about support resources, SH protocols and reporting channels is available in multiple languages.
- Using inclusive, accessible, and non-judgmental language to encourage participation from diverse audiences.
- Providing clear and practical information that is easy to understand.
- Offering multiple reporting channels, including in-person and online services.
- Improving accessibility through elements such as audio descriptions, images, and graphics.
- Including a frequently asked questions section explaining SH protocols and reporting processes in user-friendly language.
- Organizing institutional websites so resources are easy to find and regularly updated with new laws and best practices.
- Creating regular activities, such as workshops or short courses, to share and discuss SH prevention and identification.

Regarding specific actions for other vulnerable groups, we recommend:

- Strengthening networks with women’s and feminist associations, LGBTQIA+ community representatives, ethnic and cultural organizations, and other civil society organizations.
- Organizing joint activities, such as training workshops or film series, on key dates such as November 25th or Pride Day, can facilitate this process.
- Promoting courses that use a gender and intersectional perspective that considers both the risk of SH toward women and the specific needs of other groups. It is important that this training also be directed at frontline professionals, such as technical staff from university equality units and department representatives, among others.
- Training can be delivered through tailored workshops, peer support programs, and awareness sessions co-designed with representatives of these communities to ensure relevance and cultural sensitivity.
- Integrating bystander intervention training modules that emphasize sensitivity to the specific vulnerabilities and needs of at-risk groups.

### 3.3 Tertiary prevention: reducing the (unequal) impact of SH and the risk of revictimization

Social inequalities affect both the impact of SH and the risk of revictimization, making them a priority in tertiary prevention. Recommended actions include:

- Ensuring free access to support resources, including legal guidance and psychological assistance, and applying trauma-informed approaches. Where services are limited, collaboration with external specialized resources is essential.
- Including specialized staff trained in gender and intersectional perspectives in SH case management and victim-survivor support.
- Ensuring that reporting SH does not lead to academic or professional consequences, through measures such as curricular accommodations, financial support, justified absences, and workplace adjustments. Safety plans should ensure that necessary changes affect the aggressor rather than the victim-survivor.

## 4 Conclusion

In Europe, improving the effectiveness of workplace SH prevention, including in digital environments, is increasingly urgent. Universities play a key role due to their experience in promoting gender equality, diversity, and inclusion.

Training is central to prevention, especially when it reaches the entire university community, particularly vulnerable groups. Its impact can be strengthened through complementary actions such as institutional partnerships, awareness campaigns, and equality plans, addressing not only SH cases but also their root causes and consequences.

As with other forms of violence, social inequalities shape exposure, access to support, and outcomes. Addressing these inequalities across all levels of prevention is essential to effectively cope with SH in universities. In this context, the growing European commitment to tackling SH may further support and reinforce institutional efforts in higher education.
